# An Entomopathogenic Nematode by Any Other Name

**DOI:** 10.1371/journal.ppat.1002527

**Published:** 2012-03-01

**Authors:** Adler R. Dillman, John M. Chaston, Byron J. Adams, Todd A. Ciche, Heidi Goodrich-Blair, S. Patricia Stock, Paul W. Sternberg

**Affiliations:** 1 Howard Hughes Medical Institute, Division of Biology, California Institute of Technology, Pasadena, California, United States of America; 2 Department of Entomology, Cornell University, Ithaca, New York, United States of America; 3 Department of Biology, and Evolutionary Ecology Laboratories, Brigham Young University, Provo, Utah, United States of America; 4 Department of Microbiology and Molecular Genetics, Michigan State University, East Lansing, Michigan, United States of America; 5 Department of Bacteriology, University of Wisconsin-Madison, Madison, Wisconsin, United States of America; 6 Department of Entomology, University of Arizona, Tucson, Arizona, United States of America; The Fox Chase Cancer Center, United States of America

## Introduction

Among the diversity of insect-parasitic nematodes, entomopathogenic nematodes (EPNs) are distinct, cooperating with insect-pathogenic bacteria to kill insect hosts. EPNs have adapted specific mechanisms to associate with and transmit bacteria to insect hosts. New discoveries have expanded this guild of nematodes and refine our understanding of the nature and evolution of insect–nematode associations. Here, we clarify the meaning of “entomopathogenic” in nematology and argue that EPNs must rapidly kill their hosts with the aid of bacterial partners and must pass on the associated bacteria to future generations.

## Strangers, Acquaintances, and Enemies

**Figure 1:**
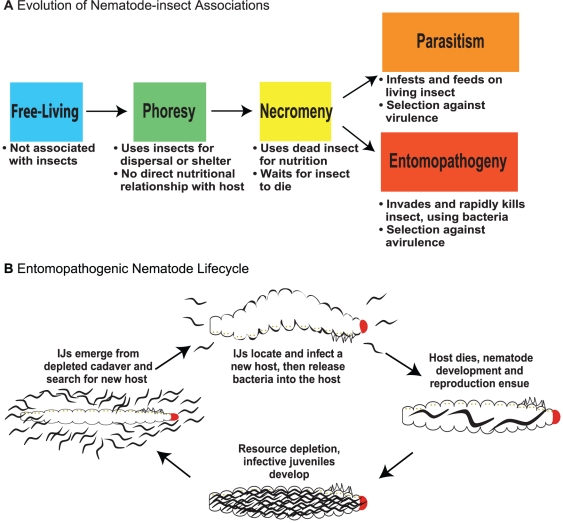
Evolution of nematode–insect associations and the entomopathogenic nematode life cycle. (A) The evolution of nematode–insect associations. **Free-living**: microbotrophic nematodes not known to associate with arthropods, vertebrates, plants, or fungi; only perhaps transiently associated with insects. **Phoresy**: a relationship where nematodes are adapted to use insects for dispersal or shelter but have no direct nutritional relationship to them. **Necromeny**: a relationship where nematodes are adapted to use saprophytic insect cadavers as a food resource but do not participate in insect death. **Parasitism**: a relationship where nematodes are adapted to use living insects directly for nutrition, likely inflicting some level of harm or even causing eventual death of the host. **Entomopathogeny**: a relationship where nematodes cooperate with insect-pathogenic bacteria to cause rapid insect disease and death and then feed and develop on the insect and bacterial resources. The distinction between parasitism and entomopathogeny is based on salient features including use of pathogenic bacteria and direction of selection (against virulence or avirulence), either making the nematodes more or less immediately harmful to their host. (B) The life cycle of entomopathogenic nematodes. The IJ stage is a developmentally arrested third larval stage and is the only free-living stage; all other stages exist exclusively within the host. EPN IJs carry symbiotic bacteria and search for potential insect hosts. They enter a host, gain access to the hemolymph, and release their bacterial symbiont. The symbiont plays a critical role in overcoming host immunity. The nematodes develop and reproduce in the resulting nutrient-rich environment until population density is high and resources begin to deplete, at which point new IJs develop and disperse, carrying the symbiotic bacteria to new hosts [5].

Nematode–arthropod associations are plentiful and range from beneficial to antagonistic [1], [2]. These associations have been divided into at least four categories: 1) phoretic (nematodes are transported by an insect), 2) necromenic (nematodes obtain nutrition from insect cadavers), 3) facultative parasitism, and 4) obligate parasitism (see Sudhaus 2008 for a more detailed breakdown [3]). It is thought that insect parasitism evolves in this sequence, with parasites evolving from non-parasitic insect associates (Figure 1A) [1], [3]. Nematodes also interact with bacteria in at least three ways: 1) trophism (nematodes eat bacteria), 2) parasitism (pathogens cause nematode diseases if not resisted), and 3) mutualism (nematodes and bacteria cooperate). Here, we consider entomopathogenic nematodes, which employ bacteria to kill insects.

## Entomopathogenic Nematodes

The term entomopathogenic is widely used in parasitology and pathology, usually referring “to microorganisms and viruses capable of causing disease in an insect host” [4]. Nematodes in Steinernematidae and Heterorhabditidae associate with pathogenic bacteria to kill insect hosts, usually within 48 hours of infection. The hallmarks of this specific type of parasitism by nematodes, known as entomopathogeny, are 1) carriage of pathogenic bacteria by infective juvenile (IJ) nematodes (also known as dauer juveniles); 2) active host-seeking and -penetration by IJs; 3) release of the bacteria into the insect hemolymph; 4) death of the insect, and nematode reproduction and bacterial proliferation driven by cadaver-nutrient utilization; 5) reassociation of the pathogenic bacteria with new generations of IJs; and 6) emergence of IJs from the nutrient-depleted cadaver as they search for new insect hosts (Figure 1B) [5], [6]. Nematode parasites of this kind are known as EPNs.

Recently, other nematode species have been shown to use pathogenic bacteria to parasitize insect hosts. Two *Oscheius* species, *Oscheius chongmingensis* and *Oscheius carolinensis*, and *Caenorhabditis briggsae* have been identified as potential insect pathogens by baiting soil for nematodes using insect larvae as prey, a common approach used for finding EPNs [7]–[11]. All of these have been found to associate with insect pathogenic bacteria of the genus *Serratia*, while *O. carolinensis* may have additional associates [9]–[12]. *O. chongmingensis* and *C. briggsae* require their bacterial partners to cause host death, and to grow and reproduce within killed insects, and emerging dauer juveniles are associated with the vectored pathogen [10], [11]. Ongoing studies suggest that these species are EPNs, though their classification as entomopathogens has been contested both semantically and conceptually in the literature and scientific meetings (e.g., the November 2010 NemaSym NSF RCN meeting and the July 2011 Society of Nematologists meeting) [13]–[15].

## History, Context, and Formal Criteria

The term entomopathogenic first appeared in the nematology literature in reference to the bacterial symbionts of *Steinernema* and *Heterorhabditis* [16]. Bacteria are considered entomopathogenic when their LD_50_ is <10,000 cells injected into the hemocoel [17]. Some pathogens associated with *Steinernema* and *Heterorhabditis* have LD_50_s<10 cells when injected, but this varies with different hosts and these bacteria are not known to infect insects without the aid of their nematode partners [18]. “Entomopathogenic” was applied to nematodes in 1981 and again in 1986 [19], [20], a use that gained momentum in 1988 [21]. This gradual, social use of the term entomopathogenic without formal definition complicates its application to emerging nematode–bacteria partnerships. Indeed, the convenience of this descriptor is currently that it applies to both partners as a complex, rather than only the nematodes or bacteria. The only clearly identifiable EPN definition that we are aware of was proposed informally [4], [22]. This definition focuses on mutualism with bacteria and on the exclusivity of the IJ as the free-living stage. We find the use of these criteria incomplete since they do not consider rapid death, which is necessary to differentiate EPNs from phoretic, necromenic, or other less virulent forms of parasitism, and the inclusion of a stage-specific requirement in defining EPNs is unnecessary. Since convention provides no standard to assess classification of EPNs, and because “entomopathogenic” was meant to differentiate insect-parasitic nematodes that serve as vectors of bacteria and to reinforce the link between nematology and insect pathology [2], we formally suggest two criteria: 1) the nematodes use a symbiotic relationship with bacteria to facilitate pathogenesis, which implies that the association is non-transient, though not necessarily obligate, and 2) insect death is sufficiently rapid that it can be unequivocally distinguished from phoretic, necromenic, and other parasitic associations (i.e., <120 h), a time frame that also implies efficient release of the pathogen by the nematode vector [17]. These criteria are based on early investigations of EPNs and what we consider the fundamental principles of the EPN lifestyle [1], [2]. We intend this discussion to provide a more thorough evaluation of the defining characteristics of EPNs, though our criteria overlap with, but are not as restrictive as, the previous definition [4], [22].

Koch's postulates can be used to establish pathogenicity of the nematode–bacterium complex or either partner alone, and we suggest that partner association across generations is particularly important in this evaluation [23]. To establish genetic heritability, genes must be passed through the F1 generation to the F2 generation; for example, a mule inherits, but does not pass on, traits inherited from its paternal donkey and maternal horse parents. Similarly, we argue that for an EPN association to be stable, nematodes must not only infect and kill an insect and produce progeny, but must also produce progeny that depart the carcass carrying the pathogenic bacteria. This does not require that the association be obligate—subsequent generations that thrive in non-insect environments may lose the symbiotic bacteria—but we believe it is crucial that symbiont transmission from the infecting parental generation to emerging nematodes from at least two subsequent insect infections be clearly established to distinguish nematode carriage of the bacteria or bona fide association from transient cuticle hitchhiking. Also, in associating, each partner must benefit from the association. At a minimum, the bacteria should increase overall nematode fitness by assisting in insect killing, nutrient liberation, or scavenger deterrence, and the nematodes should provide the bacteria with access to the insect host either by delivery to otherwise inaccessible host cavities or tissues, or by increasing dispersal range through direct carriage. Though EPNs must be capable of infecting and killing insect hosts, this does not preclude them from also, opportunistically, acting as scavengers or from competing with other EPNs for already killed insects [24], [25]. An additional cautionary point here is that the symbiont transmission rate and the stability of nematode–bacterium associations themselves have been well characterized in representative taxa [26], [27], but these details are unclear in most of the 75 EPN species reported to date [7].

Insect host killing within five days of infection is an appropriate requirement and implies selection for virulence or at least selection against avirulence, differentiating entomopathogeny from other forms of parasitism such as those used by mermithids and allantonematids. “Potentially pathogenic” bacteria, microbes that cause septicemia at low inocula when in the hemocoel but that lack mechanisms for actively invading the hemocoel [17], usually cause death within two to four days in common laboratory larvae such as *Galleria mellonella*, though larger or adult insect hosts, such as mole crickets or *Manduca sexta*, take longer to succumb, depending on the size of the nematode founding population and which pathogenic bacterium is used [18]. Rapid death caused by EPNs reflects pathogenicity of the bacterial partner with possible contributions from the nematode and relies on efficient release of the bacteria into the hemolymph.

## Specialization of EPNs

When considering appropriate criteria that define EPNs, it is tempting to use the particular details that are known for only a few representative taxa. Instead, we avoided specifics in favor of fundamental principles that underlie the associations, and observed that many interesting and often dogmatic EPN characteristics are less widespread than we expected. For example, specialization with particular bacteria is a hallmark EPN characteristic, and monospecificity between one nematode and one genus of bacteria or even one symbiont species is commonly observed among these taxa [7]. However, growing evidence of promiscuous relationships between EPNs and their bacterial symbionts suggests that this may not be as common as originally thought (e.g., [28]–[30]). Although most *Heterorhabditis* and *Steinernema* symbionts localize to the nematode intestine, there are excellent examples of nematode–bacteria symbioses in other body sites (e.g., [31]). Of note, *Paenibacillus nematophilus* associates on the cuticle of *Heterorhabditis* spp., and, relevant to this discussion, *O. carolinensis* is associated with insect pathogenic *Serratia marcescens* on its exterior cuticle [12], [30]. Also, dogma dictates that these associations are obligate, since *Steinernema* and *Heterorhabditis* symbionts are generally not free-living, and *S. carpocapsae*'s symbiont is auxotrophic for nicotinic acid, which is not available in the environment [32]. However, *Photorhabdus asymbiotica* may be free-living (e.g., [33]). Also, most nematodes require their symbionts for growth and reproduction, but exceptions have been observed (e.g., [34], [35]). There are also differences between biological characteristics of the two nematode taxa. For example, *Heterorhabditis* maternally transmit symbionts by a sophisticated multistep process, while *Steinernema* have specialized host structures within which they carry their symbionts [28], [29]. Also, some *Steinernema* infect and kill insect hosts even in the absence of pathogenic bacteria, at least in laboratory conditions, but *Heterorhabditis* nematodes have not been reported to have this behavior. Finally, as we mentioned above, symbiont transmission to new generations varies widely in the few taxa where it has been studied from >95% to ∼10% [35], [36]. Together, these findings reveal that *Steinernema* and *Heterorhabditis* are highly adapted to entomopathogeny and showcase adaptations likely to emerge as a result of long-term commitment to the entomopathogenic lifestyle, even though the biological basis for their symbiotic association with bacteria differs significantly [5], [37]. The exceptions and differences that have been observed for all of these hallmark characteristics highlight why specializations should not be used to exclude newly described associations, and emphasize that applying observations from a few representative members to whole clades can be problematic. Indeed, few species in either genus have been thoroughly explored, and we caution against assuming a priori these specializations to be true of all or even most steinernematids or heterorhabditids (e.g., [38]).

## Classification of Newly Described Associations

According to the standards we propose above, *C. briggsae* may not be an EPN. IJs recovered from dead insects seem able to reinfect new hosts but are less virulent in *G. mellonella* as a complex than injection of the bacteria alone, suggesting either inefficient release of the pathogen or some antagonism by the nematode vector. This may reflect that *C. briggsae* is somewhere between necromenic and entomopathogenic, that it is a nascent entomopathogen and not yet efficient, or that *G. mellonella* is a poor host. However, symbiont heritability has not been demonstrated, and the nature of *C. briggsae*'s bacterial association remains unresolved [10], [11], [39]. Because *C. briggsae* has not met the suggested criteria, it should not be considered an EPN, facultative or otherwise, until heritability of the pathogenic bacteria is demonstrated and more is known about bacterial release and speed of host death. Our suggested criteria have been tested and met for both *O. chongmingensis* and *O. carolinensis* [9], [10], [12]. Therefore, these taxa should be considered EPNs even though further research is required to determine the nature and heritability of their bacterial associations, and whether they are obligate or facultative EPNs.

## Symbiosis and Entomopathogeny

Nematode–bacterium partnerships that do not explicitly fulfill the requirements to be classified as EPNs are still of extraordinary interest since they may represent developing, nascent partnerships, but they should not be considered entomopathogens. Our understanding of parasitism and its evolution is continually refined as biodiversity is explored and ecology and evolution become increasingly emphasized among established and satellite model systems. We have suggested specific and restricted use of the term entomopathogenic in nematology, which will facilitate unambiguous communication. Among the 20 or more parasitic lineages of nematodes, entomopathogeny is a unique type of insect parasitism not found among vertebrate- or plant-parasitic nematodes. Recent work indicates that entomopathogeny has arisen at least three times within Nematoda, and that recently described species (*O. chongmingensis* and *O. carolinensis*) may represent nascent stages of EPN evolution. These developments emphasize the tremendous specialization exhibited by *Heterorhabditis* and *Steinernema* and increase their usefulness as models for the evolution of symbiosis and parasitism.
